# Jellyfish Stings Trigger Gill Disorders and Increased Mortality in Farmed *Sparus aurata* (Linnaeus, 1758) in the Mediterranean Sea

**DOI:** 10.1371/journal.pone.0154239

**Published:** 2016-04-21

**Authors:** Mar Bosch-Belmar, Charaf M’Rabet, Raouf Dhaouadi, Mohamed Chalghaf, Mohamed Néjib Daly Yahia, Verónica Fuentes, Stefano Piraino, Ons Kéfi-Daly Yahia

**Affiliations:** 1 Dipartimento di Scienze e Tecnologie Biologiche ed Ambientali, Università del Salento, Lecce, Italy; 2 Research group of Oceanography and Plankton, National Agronomic Institute of Tunis, Tunis, Tunisia; 3 Ecole Nationale de Médecine Vétérinaire, Sidi Thabet Ariana, Tunisia; 4 Institut Supérieur de Pêche et d’Aquaculture-Bizerte, Bizerte, Tunisia; 5 Faculté des Sciences de Bizerte, Jarzouna, Tunisia; 6 Institut de Ciències del Mar, ICM-CSIC, Barcelona, Spain; 7 CONISMA, Consorzio Nazionale Interuniversitario per le Scienze del Mare, Roma, Italy; Deakin University, AUSTRALIA

## Abstract

Jellyfish are of particular concern for marine finfish aquaculture. In recent years repeated mass mortality episodes of farmed fish were caused by blooms of gelatinous cnidarian stingers, as a consequence of a wide range of hemolytic, cytotoxic, and neurotoxic properties of associated cnidocytes venoms. The mauve stinger jellyfish *Pelagia noctiluca* (Scyphozoa) has been identified as direct causative agent for several documented fish mortality events both in Northern Europe and the Mediterranean Sea aquaculture farms. We investigated the effects of *P*. *noctiluca* envenomations on the gilthead sea bream *Sparus aurata* by *in vivo* laboratory assays. Fish were incubated for 8 hours with jellyfish at 3 different densities in 300 l experimental tanks. Gill disorders were assessed by histological analyses and histopathological scoring of samples collected at time intervals from 3 hours to 4 weeks after initial exposure. Fish gills showed different extent and severity of gill lesions according to jellyfish density and incubation time, and long after the removal of jellyfish from tanks. Jellyfish envenomation elicits local and systemic inflammation reactions, histopathology and gill cell toxicity, with severe impacts on fish health. Altogether, these results shows *P*. *noctiluca* swarms may represent a high risk for Mediterranean finfish aquaculture farms, generating significant gill damage after only a few hours of contact with farmed *S*. *aurata*. Due to the growth of the aquaculture sector and the increased frequency of jellyfish blooms in the coastal waters, negative interactions between stinging jellyfish and farmed fish are likely to increase with the potential for significant economic losses.

## Introduction

In recent years, negative interactions between jellyfish blooms (JB) and marine finfish aquaculture have been reported. Such interactions have included mass fish mortalities with severe economic impacts on the aquaculture companies [1,2]. Jellyfish can enter fish cages either intact or broken up into tentacles and other body fragments pushed by currents and waves washing in through the net cages [3,4]. Several species of cnidarian jellyfish have been reported to affect marine farmed fish of inducing skin lesions and gill damage caused by nematocyst discharge and venom injection usually leading to local inflammatory response, cell toxicity and histopathology [2,3,5]. Prolonged nematocyst discharges in fish tissues may often lead to secondary bacterial infections and associated systemic reactions, including respiratory and osmoregulatory distress, altered behaviour, and death [2,6–8]. In particular, gills have vital roles, being the main site of gas exchange, osmoregulation, acid-base balance, and excretion of nitrogen compounds [9]. Gill disorders have become one of the most serious causes of mortality in marine farmed salmon in Ireland, with average losses of 12% per year [6].

The scyphomedusa *Pelagia noctiluca* (Forsskål, 1775), also known as mauve stinger, is one of the most common stinging jellyfish species across the Eastern Atlantic and the Mediterranean Sea, producing major outbreaks with subsequently highly negative impacts on human activities, including caged finfish aquaculture [10,11]. On the Mediterranean Spanish coast, *P*. *noctiluca* is responsible for gill damage on the marine farmed fish *Dicentrarchus labrax*, leading to reduction of fish growth rate and even death [12]. Additional fish mortality events related to *P*. *noctiluca* abundance have also been recorded in Tunisian facilities (unpublished data). In 2007, a widespread occurrence of mauve stingers were documented in Irish coastal and shelf waters and caused several hundred thousand salmon mortalities [13,14]. Since then there have been several other large fish kills in UK and Irish waters [15,16]. In the same region, a bloom of moon jellyfish *Aurelia aurita* was responsible for a significant salmon mortality in summer 2010 [14,17]. Other jellyfish have also been identified as potentially harmful species for aquaculture facilities, such as the hydromedusae *Solmaris corona* and *Phialella quadrata* [3], and the siphonophore *Muggiaea atlantica* that caused the death of > 100,000 farmed fish in Norway [18]. Previous studies demonstrated also that some jellyfish species—such as *P*. *quadrata* and *P*. *noctiluca*—can act as vectors of *Tenacibaculum maritimum*, the causative agent of tenacibaculosis, a major bacterial disease affecting fish mariculture worldwide, which heavily exacerbates the impacts of jellyfish sting envenomations [19–22].

Impacts of low to medium jellyfish abundances usually remain unnoticed by aquaculture farmers and low incidence of unspecific pathologies are labelled as unknown "water borne irritant damage" [15]. However, substantial gill disorders to produce low-level mortalities might be potentially correlated also to low jellyfish abundances (Baxter el al. 2011).

Much work has been carried out on the impacts of jellies on farmed salmon aquaculture in Northern European waters [3,6,23,24]. Comparatively, little or no information is available about the impacts of one of the most harmful European jellyfish species, *P*. *noctiluca*, on the commonest Mediterranean finfish aquaculture species, such as the sea bass *D*.*labrax* and the gilthead sea bream *Sparus aurata* (Linnaeus, 1758). Due to its high adaptability to intensive rearing conditions, *S*. *aurata* represents one of the most suitable species for cultivation in ponds and marine cages, leading to the most important fish production in the Mediterranean Sea, reaching near 160.000 tonnes in 2012 [25]. In parallel, overproduction led to cutbacks in market price, calling for further reduction of production costs.

To increase knowledge on impacts of gelatinous plankton blooms on Mediterranean caged fish species and support early monitoring of risks for aquaculture production, an experimental assay was set up to assess [I] the potential histopathological damage that *P*. *noctiluca* jellyfish tissue fragments produce on gills of cultured *S*. *aurata*, [II] the impacts of different jellyfish densities on cultured fish health, and [III] the histological evolution of gill lesions over time following initial jellyfish sting treatment.

## Material and Methods

This study was performed in accordance with the European Commission Directive 2010/63/EU. The experimental protocol was designed to comply with the European policy of the “3 Rs” (Reduce, Refine, and Replace) in aquatic animal experimentation and was approved by the Institut Supérieur de Pêche et d'Aquaculture de Bizerte (Research unit 05/ur/11-15), which is under the double supervision of the Tunisia’s Ministry for Agriculture and the Hydraulic resources, and of the Ministry for Higher education and the Scientific Research and Technology.

Fish were monitored daily (early in the morning and during afternoon) over the complete experiment duration. Check-list including different humane endpoints was revised at group and also at individual level when necessary. The main established criteria were swimming behavior, skin pigmentation, frequency of opercular movements, ability of food uptake, weight loss, prostration, hyper-excitability and itching. The maintenance of animals during the experiment as well as the euthanasia procedure was monitored and carried out by trained and competent staff, in order to minimise animals’ suffering.

### Animals’ maintenance and experimental setup

A total number of 136 *Sparus aurata* adult fish (mean weight of 200 ± 19.23 g) were obtained from “Tunisian Teboulba Fish” aquaculture facility and transported to the Institut Supérieur de Péche et d'Aquaculture de Bizerte, Tunisia (ISPA). Fish were homogeneously distributed in 8 circular tanks of 300 litres each (fish stocking density of around 9 kg m^-3^) and allowed to acclimate for one week before starting the experiment. All tanks were supplied by a continuous flow (renewal rate of 23 l h^-1^) of double-filtered (5-μm, 1-μm mesh) seawater (FSW). The water circulation flow was kept at natural sea temperature of 15.5 ± 1.0°C and 36.8 ± 0.3 salinity) with aeration to keep dissolved oxygen at 100% saturation. Throughout the experiment, the fish were fed daily with standard commercial pellets (Skretting S.A.) and maintained under a natural photoperiod (12 h light, 12 h dark).

Jellyfish (4.5 ± 0.9 cm bell diameter) were collected by a dip net the day before the start of the experiment from the Channel of Bizerte (Tunisia) and maintained in 25 litres buckets with FSW and at low density for one day. *Pelagia noctiluca* jellyfish is not an endangered or protected species. Specimens from Bizerte gulf were collected without the need of a permit because sampling was never conducted in a restricted marine area.

To simulate a realistic encounter between jellyfish that had been pressed by currents against aquaculture cages and cultured fish, jellyfish were chopped into small (≥ 1 cm) pieces immediately prior to the start of the jellyfish exposure. The four treatment groups consisted of two control tanks (without jellyfish) and six tanks with chopped *P*. *noctiluca* at low (LJ), medium (MJ), and high jellyfish densities (HJ): 3, 7 and 15 jellyfish per tank with 18 experimental fish (10, 25 and 50 jellyfish m^-3^, approximately equivalent to 350 g, 875 g and 1750 g jellyfish biomass, respectively). These densities were predetermined to reproduce a range of different jellyfish concentrations observed during *P*. *noctiluca* bloom periods in Tunisian waters and Sicily Channel (unpublished observations). A 1-mm stainless steel mesh was placed at the outlet of each tank preventing jellyfish pieces to spill out the experimental tanks.

The experiment began when jellyfish pieces were placed simultaneously in all treatment tanks with fish. The maximum fish-jellyfish interaction lasted 8 h; after that, all jellyfish pieces were removed using a 200-μm mesh hand net. The exposure time to jellyfish tissue of 8 h was used to represent the minimum night time with *P*. *noctiluca* jellyfish in surface waters, following sunset and the diel vertical migration of their crustacean prey [26–28].

Fish health was monitored nine times during the experiment: shortly before jellyfish incorporation to the fish tanks (0 h), during fish-jellyfish contact (3h), one hour after the removal of the jellyfish (9h), and six later times, 24 and 48h; 1, 2, 3 and 4wk, respectively before the end of the experiment at 4 weeks. At the highest jellyfish density sampling was not carried out at 24 h, 3 and 4 weeks because of the shortage of experimental individuals and fish mortalities. At each sampling time, 4 fish were randomly sampled from each treatment group (two per tank), anesthetised and then killed according to the current animal care rules using a lethal dose of UNICAINE 2% (lidocaine-HCl 500 ppm) [29]. Immediately after death, which occurred within 2–3 minutes of anaesthetic application, fish were weighed and measured, and their skin and gills visually examined for gross pathology, such as scale loss, excess mucus, pale gill filaments, swelling, necrosis and the presence of macro-parasites [30]. Two gill arches were excised from each fish and immediately preserved in 10% neutral buffered formalin for histological analysis. Tissues then were embedded in paraffin, cut by microtome into 2–5 μm sections and stained following a standard haematoxylin-eosin protocol. For each gill arch, several sections were examined microscopically at 100X and 400X magnifications.

### Gill score protocol

Interpretation of the gill damage was based on a recently developed gill histopathology scoring system [4,12], rating the potential damage on each gill sample by a total score ranging from 0 to 24, obtained by summation of partial scores assigned to different primary and secondary criteria. Primary parameters were related to 3 specific pathologies: epithelial hyperplasia (increased cell production), lamellar fusion, and cellular anomalies (degeneration, necrosis and sloughing). According to the presence, extent and severity of those pathologies, primary scores ranged from 0 to 3. In addition, a 0 or 1 score was attributed to the absence or presence of each of the following secondary parameters: hypertrophy, oedema, eosinophilic granular cells, inflammation, circulatory damage, congestion, bacterial pathogens and parasitic pathogens. The total score assigned for primary and secondary parameters, allowed classification of fish gill damage according to four cumulative score ranges: 0–3 = no significant pathology, 4–6 = mild gill pathology of minor clinical significance, 7–9 = moderate gill pathology of clinical significance, ≥ 10 = severe gill pathology of high clinical significance.

### Statistical analysis

A Shapiro-Wilk test indicated that the assumptions of normality were violated (p < 0.05, SPSS v. 20.0); therefore, differences among treatments and among sampling weeks were tested using the non-parametric one-way Kruskal-Wallis test (SPSS v.20.0). Significant results were further tested by pairwise post-hoc comparisons (Mann-Whitney U test, SPSS v. 20.0), adjusted for type I error, and Similarity percentages analysis, SIMPER (PRIMER 6).

## Results

**Fig 1:**
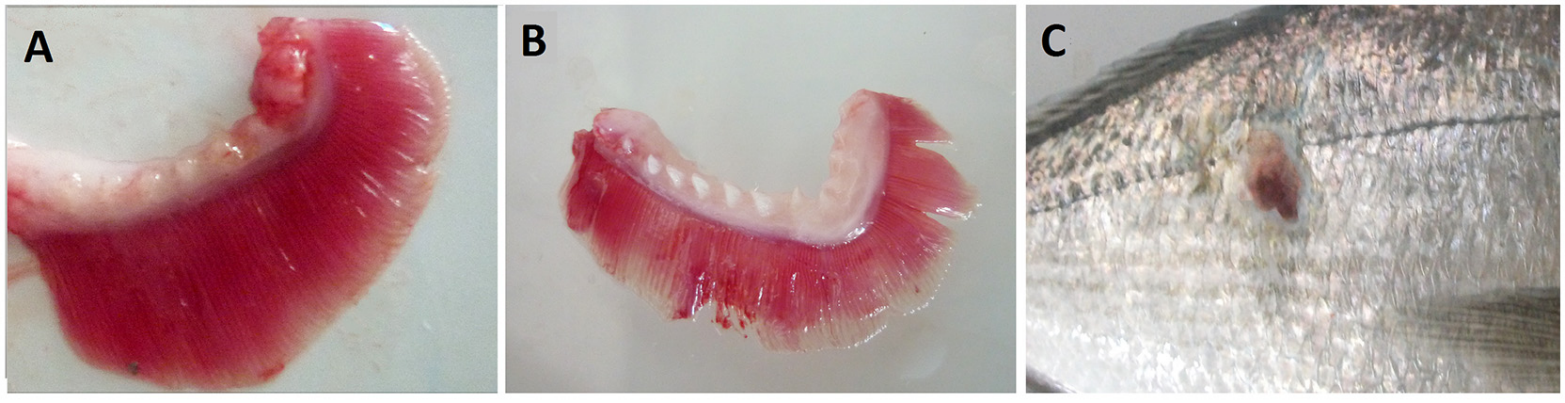
External lesions on *Sparus aurata* due to *Pelagia noctiluca* jellyfish exposure. A. Fish gill from control group; B. abrasion, haemorrhage, depigmentation and increased thickness of lamellar filaments of a fish from the high jellyfish density group 24 h after exposure to jellyfish; C. wound with necrotic tissue on the flank of *Sparus aurata* fish from the medium density group 2 weeks after exposure to jellyfish.

**Fig 2:**
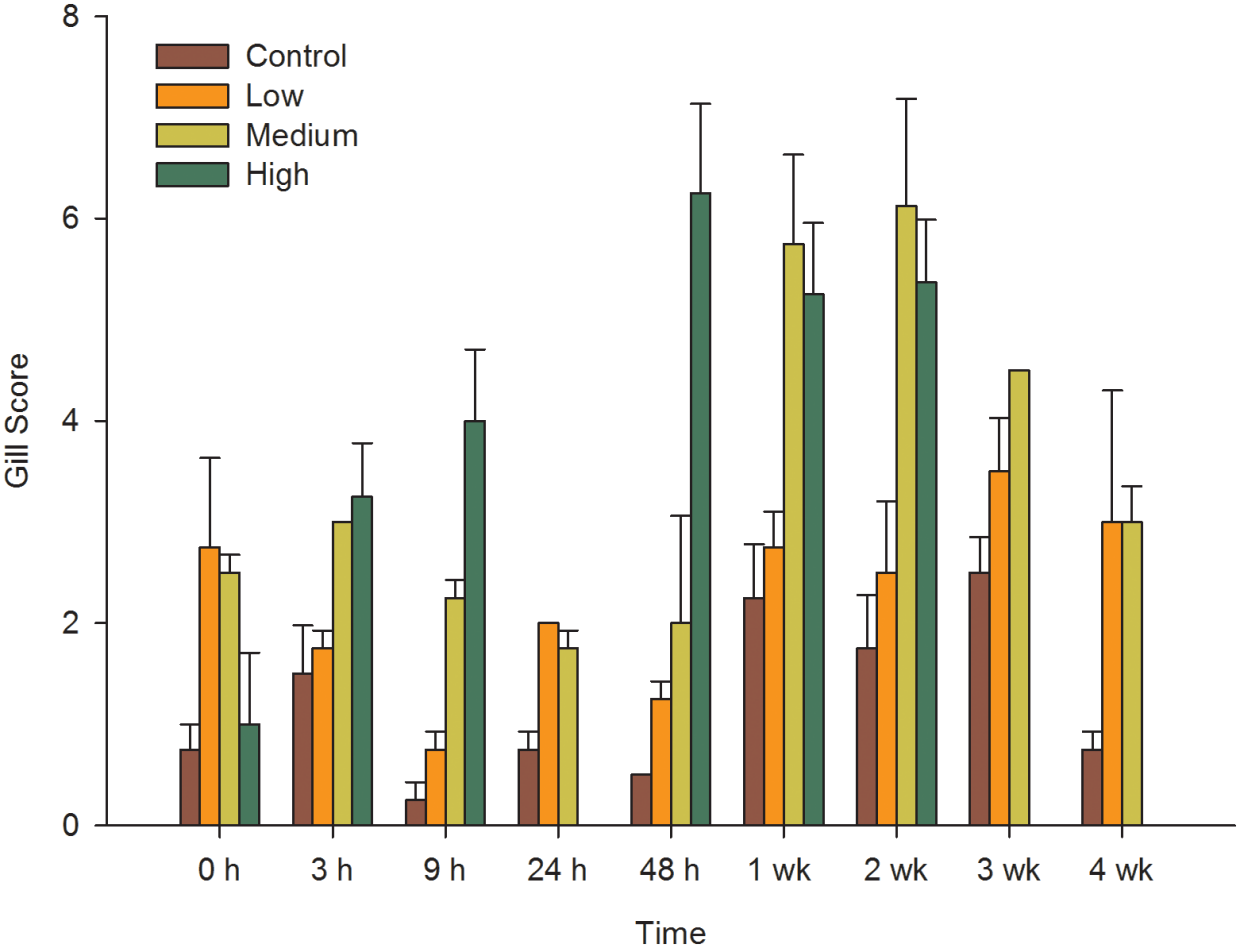
Average gill scores of treatment groups. Gill scores of control, low, medium and high *Pelagia noctiluca* jellyfish density groups before (0 h) and at different times after *Sparus aurata* exposure to jellyfish. Fish were not sampled from the highest jellyfish density group at 24 h, 3 and 4 weeks sampling points (vertical bars denote standard error).

Gills from the control fish group without jellyfish retained a normal morphology throughout the experiment. Each gill arch supported many distinct and regular filaments arranged perpendicularly in two rows and without significant lesions. In contrast, gross pathology in fish exposed to jellyfish pieces was observed throughout the experiment (Fig 1), with the extent and intensity of gill damage increasing with time and jellyfish density (Fig 2).

At 3 h after initial contact with jellyfish pieces, fish gills already showed abrasion of lamellar filaments (Fig 1A). After 24 h from the exposure to jellyfish, depigmentation, increasing thickness of lamellar filaments and haemorrhage in gill tissue were also recorded. Mild epitheliocystis [31,32] was observed in control and treated fish through the identification of spherical cysts that were circumscribed by an eosinophilic hyaline capsule. One day before the start of the experiment (24 h after the exposure to jellyfish), snout irritation, scale loss on the flanks and damage in the caudal and dorsal fins and operculum were also observed in fish in the medium and high jellyfish density groups (Fig 1B). Respiratory distress, jumping and swimming near the water surface were also observed for some treated fish throughout the exposure period to jellyfish at different jellyfish densities. A slight trend of weight reduction was observed in treated fish, possibly due to the ceased feeding behaviour observed through the experiment, but no significant statistical differences were found in weight or length analysis.

The histopathological analysis showed that the lowest gill damage score was in the control group, characterised by low levels of lamellar hyperplasia and occasional fusion, a background level of pathology typical of marine-farmed fish [6]. Gill scores from the control group were significantly different (lower) than all the groups with jellyfish (U_1_ = 25.267, p = 0.001). Gill scores also differed significantly among the groups treated with jellyfish (U_2_ = 7.050, p = 0.029). The gill scores in the LJ density group showed no significant differences throughout the experiment (U_8_ = 12.604, p = 0.126), with average scores of 2.25 ± 0.9 (SE). For the MJ density group, significant gill lesions were observed 1 week after the start of the experiment (U_1_ = 4.86, p = 0.027), with scores peaking after 2 weeks (gill score 6 ± 1.5 SE). Significant gill damage was observed immediately in the HJ density group, only 3h after the exposure to jellyfish began (U_1_ = 4.513, p = 0.034). Those high scores continued over time with a peak after 48h (6 ± 1.3 SE) (Fig 2)

**Fig 3:**
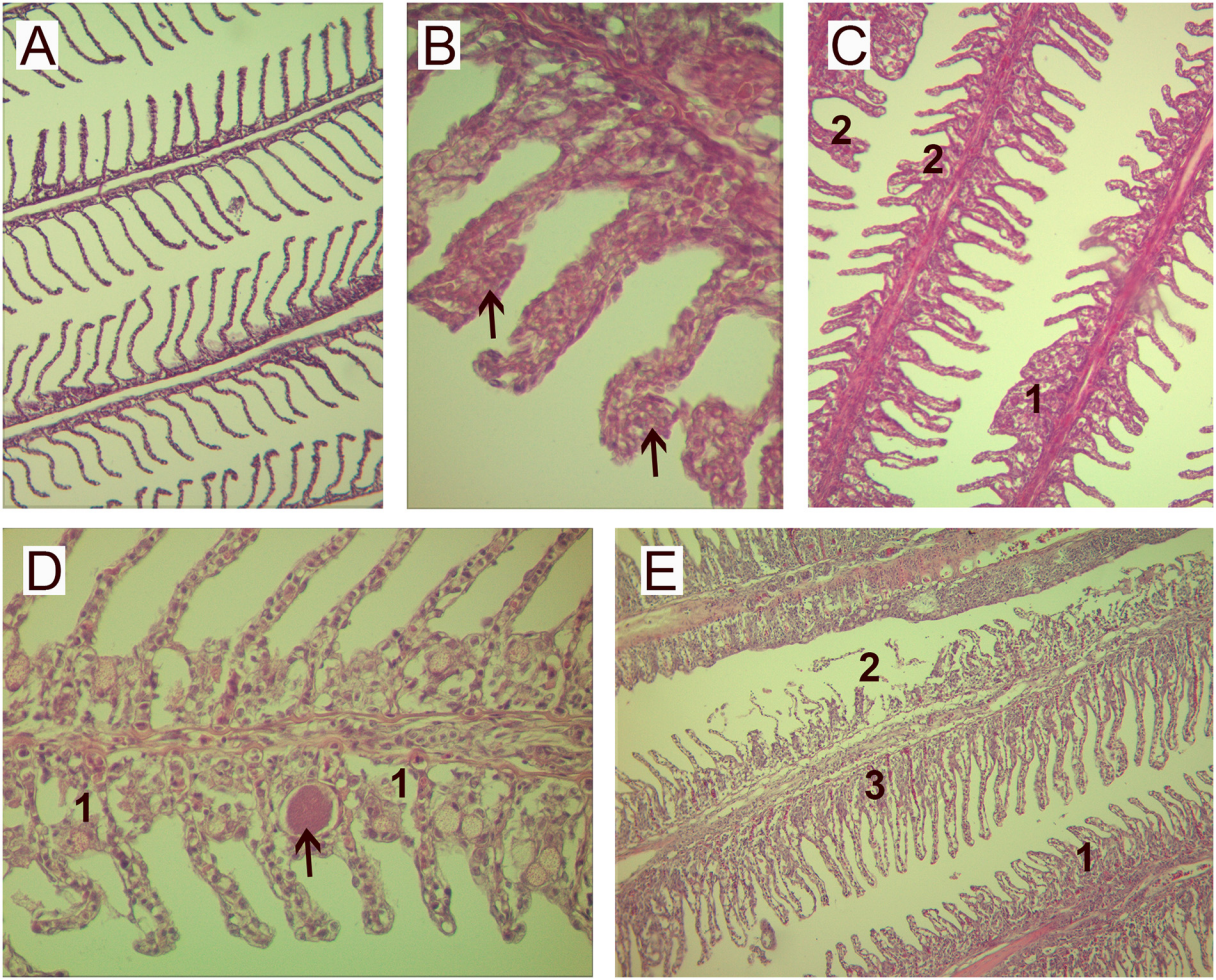
Gill lesions in fish exposed to *Pelagia noctiluca*. A. Healthy fish gill from the control (no jellyfish) group (0h) (100x); B-E. pathology in fish gills from the treatment groups after 8-h exposure to jellyfish: B. black arrows indicate lamellar hyperplasia on fish gill from the low jellyfish density group at 9h (400x); C. lamellar hyperplasia (1) and fusion (2) from the medium jellyfish density group after 1 week (100x); D. epitheliocystis (black arrow) and lamellar oedema (1) from the medium jellyfish density group after 3 weeks (400x); E. hyperplasia of the epithelium of the primary lamellae (1), necrosis focal of secondary lamellae (2) and circulatory disturbances (3) from the high jellyfish density group after 48h (100x).

**Fig 4:**
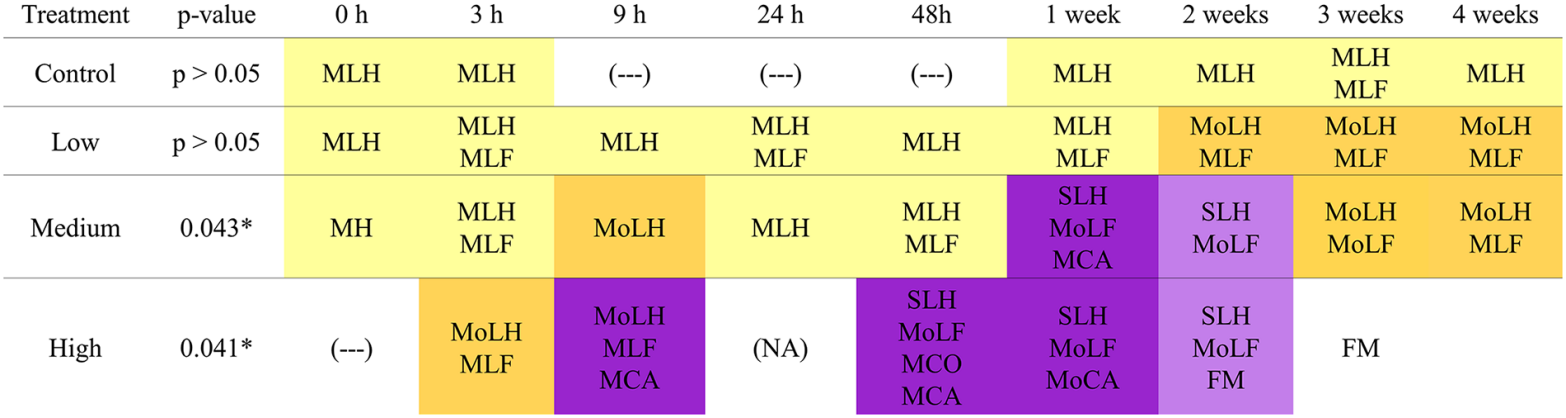
Histopatholical gill damage of experimental groups over time. MLH: Mild lamellar hyperplasia; MLF: Mild lamellar fusion; MoLH: Moderate lamellar hyperplasia; MoLF: Moderate lamellar fusion; MCA: Mild cellular anomalies; MoCA: Moderate cellular anomalies; SLH: Severe lamellar hyperplasia; MCO: Mild cellular oedema; FM: Fish mortality; (NA): data not available; (---): Non significant gill damage. Colours indicate the severity of gill damage: cream colour = mild injuries; orange = medium level of injuries; violet and purple = medium-high and high level of gill damage respectively.

Over the duration of experiment, 6 out of 136 experimental individuals died. Fish mortalities happened in the HJ density group during the second and third week of experiment, after the peak in gill damage scores. Gross pathology showed some slight external lesions mainly in fish flank. Fish showed excessive mucus production and pale gills, hyperplasia, severe lamellar fusion, desquamation, necrotic patches, lamellar congestion and lamellar oedema in some areas of the gills. Gill epithelium lesions are known to be responsible of respiratory problems and osmoregulation disorders, such as hydro-mineral equilibrium disturbances and alterations in the excretion of nitrogenous waste (NH_4_^+^). All these troubles leaded death of fish. In the MJ density group, gill scores decreased during the third and fourth week of sampling, mainly because of reduction in the percentages of hyperplasia and cellular anomalies. By contrast, fish from the LJ density group presented mild damage during the experiment, principally represented by hyperplasia and lamellar fusion (Figs 3 and 4).

The gill scores for the experimental treatment groups ranged from 1 to 9 over the entire experiment, with most fish displaying moderate lesions considered to be of clinical significance. The SIMPER analysis showed that lamellar fusion and hyperplasia were the most common lesions in all treated groups. Also, a severe inflammatory response was noted beginning at 9h after the exposure to jellyfish. The severity of gill damages was directly proportional to jellyfish density, with increasing cellular anomalies over time.

## Discussion

Frequency of occurrence and abundance of *P*. *noctiluca* vary across the Mediterranean, but dense populations can be recorded most of the year at several coastal localities, such as the channel of Bizerte (Tunisia) and the Strait of Messina (Italy) [10,33,34]. Our laboratory experiments simulated the potential consequences of blooms of the scyphomedusa *P*. *noctiluca* on finfish aquaculture farms. Our results showed that jellyfish stings can severely affect caged *S*. *aurata* fish by causing significant gill damage shortly after contact with jellyfish tissues and subsequent deterioration on fish health.

Comparable gill damage was observed previously in farmed salmon (*Salmo salar*) during blooms of *P*. *noctiluca* and *Aurelia aurita* scyphomedusae in northern Europe [6,14]. This first experimental challenge trial between fish in culture and jellyfish exposed juvenile *S*. *salar* to realistic *A*. *aurita* jellyfish bloom densities showed significant and increasing gill damage starting 24 h after the initial contact [6].

Here we investigated the intensity of gill damage on cultured sea bream at increasing *P*. *noctiluca* densities. At low jellyfish density (up to 10 jellyfish m^-3^), mild damage to fish gills were observed. Conversely, at higher jellyfish concentrations (≥ 25 jellyfish m^-3^) impacts ranged from moderate damage, leading to potential effects on the fish metabolism, to more severe consequences including death, due to high levels of lesions and respiratory distress [2].

Three weeks after the initial exposure to jellyfish, fish from the medium density group showed early signs of tissue repair in the gills. Recovery was characterized by significant decreases in the percentages of lamellar hyperplasia and fusion, in observed inflammatory reactions, and disappearance of cellular anomalies. At last, recovery of tissue integrity was observed in fish in the MJ density group, whereas fish from HJ density died 2–3 weeks after exposure to jellyfish. Exposure to HJ density led to intense and increasing gill damage, eventually impairing homeostatic mechanisms and adaptive physiological responses [35]. Non bacterial infection of *Tenacibaculum* sp. was confirmed, due to the absence of filamentous bacterial mats on the necrotic patches [36]. Overall, these results indicate that even short exposure to jellyfish can result in significant gill damage in marine-farmed fish, with potential increase in extent and severity of damage even when jellyfish are no longer present.

Our results also indicate that the potential impact of jellyfish on marine wild fish populations might not be negligible. Previous research on fish-jellyfish interactions are mostly focused on jellyfish predation on fish or, conversely, the use of jellyfish biomasses by medusivorous fish as temporary or exclusive food source [34,37–39]. The outcome of jellyfish interactions with fish populations depends on several factors affecting the probability of encounters, including water temperature, dissolved oxygen, and the size and density of predators and prey [40]. For several jellyfish species, bloom density may reach extremely high values. *Pelagia noctiluca* in the Mediterranean Sea occurs in large swarms reaching densities over 100 medusae m^-3^ for prolonged periods (up to weeks), with temporary aggregations caused by wind, currents, coastal geomorphology and jellyfish behaviour containing up to 600 medusae m^-3^ [41,42]. These values largely exceed the experimental density values used in our fish-jellyfish interaction experiments (10, 25, 50 medusae m^-3^). Furthermore, shortly after sexual reproduction—in springtime—large swarms of ephyrae and juvenile jellyfish are regularly encountered in the Southern Tyrrhenian Sea (Aeolian islands), with much higher densities, up to several thousands of individuals m^-3^; (Piraino, pers. observation; see also https://goo.gl/G8GNl8). Temporary paramount densities may therefore represent a key threat affecting the physiological integrity and health of fish living in sheltered areas where extremely high jellyfish aggregations occur, such as bays or fjords (with records up to 1000 *Periphylla periphylla* medusae m^-3^ [43,44]).

Further investigations are required to clarify whether the potential rise of both temperature and jellyfish numbers in a global change scenario may exacerbate negative impacts not only on farmed fish, but also on wild fish populations [1,45,46].

The consequences of episodes of jellyfish proliferation can be of high importance for aquaculture, considering they could affect not only fish health, but also the growth and quality of caged fish [2,30]. The sudden and unpredictable nature of jellyfish blooms hinders the implementation of preventive measures against their negative effects in aquaculture. Because of this, the development and implementation of swift mitigation procedures are crucial and must be rooted in knowledge of the type and extent of physical damage caused by jellyfish. Even a low density of *P*. *noctiluca* jellyfish could be detrimental to the health of caged fish, causing minor but significant gill lesions, which may progress over time and be worsened by bacterial infections. Investigation of the different effects of *P*. *noctiluca* blooms will enable estimation of the response time required by aquaculture facilities to undertake appropriate countermeasures that could differ in magnitude according to the damage level. Due to the recent and projected future growth of the aquaculture sector [47] and the increased frequency of jellyfish blooms in Mediterranean coastal waters [45,48], negative interactions between stinging jellyfish and caged finfish may turn into a substantial problem with high economic losses [14].

## References

[1] Purcell JE, Uye S, Lo W-T. Anthropogenic causes of jellyfish blooms and their direct consequences for humans: a review. Mar Ecol Prog Ser. 2007;350: 153–174.

[2] Rodger HD, Henry L, Mitchell SO. Non-infectious gill disorders of marine salmonid fish. Rev Fish Biol Fish. 2011;21: 423–440.

[3] Baxter EJ, Rodger HD, McAllen R, Doyle TK. Gill disorders in marine-farmed salmon: investigating the role of hydrozoan jellyfish. Aquac Environ Interact. 2011;1: 245–257.

[4] Mitchell SO, Baxter EJ, Holland C, Rodger HD. Development of a novel histopathological gill scoring protocol for assessment of gill health during a longitudinal study in marine-farmed Atlantic salmon (*Salmo salar*). Aquac Int. 2012;20: 813–825.

[5] Helmholz H, Johnston B, Ruhnau C, Prange A. Gill cell toxicity of northern boreal scyphomedusae *Cyanea capillata* and *Aurelia aurita* measured by an in vitro cell assay. Hydrobiologia. 2010;645: 223–234.

[6] Baxter EJ, Sturt MM, Ruane NM, Doyle TK, McAllen R, Harman L, et al Gill damage to Altantic salmon (*Salmo salar*) caused by the common jellyfish (*Aurelia aurita*) under experimental challenge. PLoS One. 2011;6: e18529 10.1371/journal.pone.0018529 21490977 PMC3072396

[7] Bruno DW, Ellis AES. Mortalities in farmed Atlantic salmon associated with the jellyfish *Phialella quadrata*. Bull Eur Ass Fish Pathol. 1985;5: 1984–1985.

[8] Seaton DD. Fish kills by planktonic organisms. Aquac Inf Ser. 1989;9: 1–10.

[9] Marques dos Santos DC, Pinto da Matta SL, Alves de Oliveira J, Dergam dos Santos JA. Histological alterations in gills of *Astyanax aff*. *bimaculatus* caused by acute exposition to zinc. Exp Toxicol Pathol. 2012;64: 861–866. 10.1016/j.etp.2011.03.007 21478002

[10] Canepa A, Fuentes V, Sabatés A, Piraino S, Ferdinando B, Josep-María G. *Pelagia noctiluca* in the Mediterranean Sea In: Pitt KA, Lucas CH, editors. Jellyfish Blooms. Springer; 2014 pp. 237–266.

[11] CIESM. Gelatinous zooplankton outbreaks: theory and practice. CIESM Workshop Series. Monaco; 2001. p. 112. Available: www.ciesm.org/publications/Naples01.pdf

[12] Baxter EJ, Albinyana G, Girons A, Isern MM, García AB, Lopez M, et al Jellyfish-inflicted gill damage in marine-farmed fish: an emerging problem for the Mediterranean? XIII Congreso Nacional de Acuicultura. Barcelona; 2011.

[13] Doyle TK, De Haas H, Cotton D, Dorschel B, Cummins V, Houghton JDR, et al Widespread occurrence of the jellyfish *Pelagia noctiluca* in Irish coastal and shelf waters. J Plankton Res. 2008;30: 963–968.

[14] Purcell JE, Baxter EJ, Fuentes VL. Jellyfish as products and problems of aquaculture In: Allan G, Burnell G, editors. Advances in aquaculture hatchery technology. 1st ed Woodhead Publishing; 2013 pp. 404–430.

[15] Marcos-López M, Mitchell SO, Rodger HD. Pathology and mortality associated with the mauve stinger jellyfish *Pelagia noctiluca* in farmed Atlantic salmon *Salmo salar* L. J Fish Dis. 2014; 1–5. 10.1111/jfd.12267 24909954

[16] FIS. Jellyfish kills thousands of salmon in Scottish farm. In: Fish Information & Services. 17 Dec 2014. Available: http://www.fis.com/fis/worldnews/worldnews.asp?monthyear=12-2014&day=17&id=73474&l=e&country=&special=&ndb=1&df=1. Accessed 15 Feb 2016.

[17] Mitchell SO, Baxter EJ, Rodger HD. Gill pathology in farmed salmon associated with the jellyfish *Aurelia aurita*. Vet Rec Case Reports. 2013;1: e100045. 10.1136/vr.100045 21984565

[18] Fosså J, Flood P, Olsen A, Jensen F. Småog usynlige, men plagsomme maneter av arten *Muggiaea atlantica* (Small and invisible, but troublesome jellyfish of the species *Muggiaea Atlantica*). Fisk og Havet (Fish Sea). 2003;2: 99–103.

[19] Avendaño-Herrera R, Toranzo AE, Magariños B. Tenacibaculosis infection in marine fish caused by *Tenacibaculum maritimum*: a review. Dis Aquat Organ. 2006;71: 255–266. 17058606 10.3354/dao071255

[20] Delannoy CMJ, Houghton JDR, Fleming NEC, Ferguson HW. Mauve Stingers (*Pelagia noctiluca*) as carriers of the bacterial fish pathogen *Tenacibaculum maritimum*. Aquaculture. 2011;311: 255–257.

[21] Ferguson HW, Christian M. Delannoy CM, Nicolson J, Sutherland D, Crumlish M. Jellyfish as vectors of bacterial disease for farmed salmon (Salmo salar). J Vet Diagn Invest. 2010;22: 376–382. 20453210 10.1177/104063871002200305

[22] Toranzo AE, Magariños B, Romalde JL. A review of the main bacterial fish diseases in mariculture systems. Aquaculture. 2005;246: 37–61.

[23] Baxter EJ, Sturt MM, Ruane NM, Doyle K, Mcallen R, Rodger HD. Biofouling of the hydroid *Ectopleura larynx* on aquaculture nets in Ireland: Implications for finfish health. Fish Vet J. 2012;13: 17–29.

[24] Carl C, Guenther J, Sunde LM. Larval release and attachment modes of the hydroid *Ectopleura larynx* on aquaculture nets in Norway. Aquac Res. 2010; 1–5.

[25] Colloca F, Cerasi S. Cultured Aquatic Species Information Programme. *Sparus aurata* Cultured Aquatic Species Information Programme. Rome; 2015. Available: http://www.fao.org/fishery/culturedspecies/Sparus_aurata/en

[26] Ferraris M, Berline L, Lombard F, Guidi L, Elineau A, Mendoza-Vera JM, et al Distribution of *Pelagia noctiluca* (Cnidaria, Scyphozoa) in the Ligurian Sea (NW Mediterranean Sea). J. Plankton Res. 2012;34:874–885.

[27] Axiak V. Effect of decreasing light intensity on the activity of the scyphomedusa, Pelagia noctiluca (Forskal). UNEP: Report on the workshop on jellyfish blooms in the Mediterranean. Athens; 1984. pp. 121–127.

[28] Franqueville C. Macroplancton profond (invertébrés) de la Méditerranée nord-occidentale (Deep-sea macrozooplankton (invertebrates) in north-western Mediterranean). Tethys. 1971;3: 11–56.

[29] Park IS, Park SJ, Gil HW, Nam YK, Kim DS. Anesthetic effects of clove oil and lidocaine-HCl on marine medaka (*Oryzias dancena*). Lab Anim. 2011;40: 45–51. 10.1038/laban0211-45 21252980

[30] Mitchell SO, Rodger HD. A review of infectious gill disease in marine salmonid fish. J Fish Dis. 2011;34: 411–432. 10.1111/j.1365-2761.2011.01251.x 21401646

[31] Hoffman GL, Dunbar CE, Wolf K, Zwillenberg LO. Epitheliocystis, a new infectious disease of the bluegill (*Lepomis macrochirus*). Antonie Van Leeuwenhoek. 1969;35: 146–158. 4987372 10.1007/BF02219125

[32] Nowak BF, LaPatra SE. Epitheliocystis in fish. J Fish Dis. 2006;29: 573–588. 17026667 10.1111/j.1365-2761.2006.00747.x

[33] Rosa S, Pansera M, Granata A, Guglielmo L. Interannual variability, growth, reproduction and feeding of *Pelagia noctiluca* (Cnidaria: Scyphozoa) in the Straits of Messina (Central Mediterranean Sea): Linkages with temperature and diet. J Mar Syst. 2013;111–112: 97–107.

[34] Milisenda G, Rosa S, Fuentes VL, Boero F, Guglielmo L, Purcell JE, et al Jellyfish as Prey: Frequency of Predation and Selective Foraging of *Boops boops* (Vertebrata, Actinopterygii) on the Mauve Stinger *Pelagia noctiluca* (Cnidaria, Scyphozoa). PLoS One. 2014;9: e94600 10.1371/journal.pone.0094600 24727977 PMC3984264

[35] Ingerslev HC, Lunder T, Nielsen ME. Inflammatory and regenerative responses in salmonids following mechanical tissue damage and natural infection. Fish Shellfish Immunol. 2010;29: 440–450. 10.1016/j.fsi.2010.05.002 20472069

[36] Powell MD, Harris JO, Carson J. Effects of gill abrasion and experimental infection with *Tenacibaculum maritimum* on the respiratory physiology of Atlantic salmon *Salmo salar* affected by amoebic gill disease. Dis Aquat Organ. 2005;63: 169–174. 15819432 10.3354/dao063169

[37] Ates RML. Medusivorous fishes, a review. Zool Meded. 1988;62: 29–42.

[38] D’Ambra I, Graham WM, Carmichael RH, Hernandez FJ. Fish rely on scyphozoan hosts as a primary food source: evidence from stable isotope analysis. Mar Biol. 2015;162: 247–252.

[39] Purcell J, Arai M. Interactions of pelagic cnidarians and ctenophores with fish: a review. Hydrobiologia. 2001;451: 27–44.

[40] He W, Cao Z-D, Fu S-J. Effect of temperature on hypoxia tolerance and its underlying biochemical mechanism in two juvenile cyprinids exhibiting distinct hypoxia sensitivities. Comp Biochem Physiol Part A Mol Integr Physiol. 2015;187: 232–341. 10.1016/j.cbpa.2014.05.004 24853206

[41] Zavodnik D. Spatial aggregations of the swarming jellyfish Pelagia noctiluca (Scyphozoa). Mar Biol. 1987;94: 265–269.

[42] Malej A. Behaviour and trophic ecology of the jellyfish Pelagia noctiluca (Forsskål, 1775). J Exp Mar Bio Ecol. 1989;126: 259–270.

[43] Sornes TA, Aksnes DL, Bamstedt U, Youngbluth MJ. Causes for mass occurrences of the jellyfish Periphylla periphylla: a hypothesis that involves optically conditioned retention. J Plankton Res. 2007;29: 157–167.

[44] Youngbluth MJ, Båmstedt U. Distribution, abundance, behavior and metabolism of *Periphylla periphylla*, a mesopelagic coronate medusa in a Norwegian fjord. Hydrobiologia. 2001;451: 321–333.

[45] Brotz L, Cheung W, Kleisner K, Pakhomov E, Pauly D. Increasing jellyfish populations: trends in Large Marine Ecosystems. Hydrobiologia. 2012;690: 3–20.

[46] Byrne M, Przeslawski R. Multistressor Impacts of Warming and Acidification of the Ocean on Marine Invertebrates’ Life Histories. Integr Comp Biol. 2013;53: 582–596. 10.1093/icb/ict049 23697893

[47] FAO. The state of world fisheries and aquaculture 2014. Rome; 2014.

[48] Condon RH, Duarte CM, Pitt KA, Robinson KL, Lucas CH, Sutherland KR, et al Recurrent jellyfish blooms are a consequence of global oscillations. Proc Natl Acad Sci. 2013;110: 1000–1005. 10.1073/pnas.1210920110 23277544 PMC3549082

